# Mechanisms of Ferroptosis and Relations With Regulated Cell Death: A Review

**DOI:** 10.3389/fphys.2019.00139

**Published:** 2019-02-26

**Authors:** Pengxu Lei, Tao Bai, Yuling Sun

**Affiliations:** Department of Hepatobiliary and Pancreatic Surgery, The First Affiliated Hospital of Zhengzhou University, School of Medicine, Institute of Hepatobiliary and Pancreatic Diseases, Zhengzhou University, Zhengzhou, China

**Keywords:** ferroptosis, RCD, GPX4, lipid peroxides, lipid autoxidation, iron

## Abstract

Ferroptosis is a newly identified form of nonapoptotic regulated cell death (RCD) characterized by iron-dependent accumulation of lipid peroxides. It is morphologically and biochemically different from known types of cell death. Ferroptosis plays a vital role in the treatment of tumors, renal failure, and ischemia reperfusion injury (IRI). Inhibition of glutathione peroxidase 4 (GPX4), starvation of cysteine, and peroxidation of arachidonoyl (AA) trigger ferroptosis in the cells. Iron chelators, lipophilic antioxidants, and specific inhibitor prevent ferroptosis. Although massive researches have demonstrated the importance of ferroptosis in human, its mechanism is not really clear. In this review, we distanced ourselves from this confusion by dividing the mechanisms of ferroptosis into two aspects: processes that facilitate the formation of lipid peroxides and processes that suppress the reduction of lipid peroxides. At the same time, we summarize the relations between ferroptosis and several types of cell death.

## Introduction

Cell death is the core of most pathological processes and is also an indispensable element of the regulation of normal tissues. Scientists originally thought that there were two forms of cell death: regulated cell death (RCD) and necrosis. Caspase-dependent apoptosis was widely considered to be synonymous with RCD, but the discovery of several types of nonapoptotic RCD revealed otherwise: apoptosis-inducing factor 1 (AIF1)-dependent parthanatos, receptor-interacting protein kinase 1 (RIPK1)-dependent necroptosis, and iron-dependent ferroptosis (Bergsbaken et al., 2009; Christofferson and Yuan, 2010; Dixon et al., 2012). Actually, before ferroptosis was named, this form of cell death had already been observed *in vitro*: two groups found that two small-molecule compounds, erastin and RAS-selective lethal 3 (RSL3), selectively killed oncogenic RAS mutant cells *in vitro* (Dolma et al., 2003; Yang and Stockwell, 2008). This form of cell death differed from known forms of cell death in morphological and biochemical features. Meanwhile, this process could be prevented by iron chelators and mediated by cellular iron abundance. This is why it was named ferroptosis (Dolma et al., 2003; Yagoda et al., 2007; Yang and Stockwell, 2008; Dixon et al., 2012). Since then, researchers have gradually uncovered the mechanism of ferroptosis, demonstrating that amino acids, lipids, and oxidation–reduction reaction are involved in this process (Dixon et al., 2012; Yang et al., 2014, 2016; Kagan et al., 2017). The iron-dependent accumulation of lipid peroxides is regarded as the lethal element. The decreased reduction of lipid peroxides caused by the inhibition of glutathione peroxidase 4 (GPX4) and the increased generation of lipid peroxides from arachidonoyl (AA) are two major pathways that lead to ferroptosis. Ferroptosis plays a vital role in human and participates in the initiation and development of numerous diseases [e.g., tumorigenesis, ischemia reperfusion injury (IRI), renal failure, nervous system diseases, and hematological system diseases] (Friedmann Angeli et al., 2014; Linkermann et al., 2014; Yang et al., 2014; Yu et al., 2015). Whether ferroptosis takes part in the development of more diseases is unclear, but it is believed that ferroptosis could be a physiological process that widely occurs in the body of mammals rather than a pathological or organ-specific process. Differed from other forms of cell death, ferroptosis shares a few common features with several other RCDs (Linkermann et al., 2014; Zille et al., 2017).

## Mechanisms of Ferroptosis

Iron and lipid peroxides are two major participants in ferroptosis (Dixon et al., 2012; Yang et al., 2016; Kagan et al., 2017). It seems that the accumulation of lipid peroxides, mainly phosphatidylethanolamine-OOH (PE-OOH), ultimately results in ferroptosis (Kagan et al., 2017), while iron appears to serve as a catalyst or a component of a key regulator of ferroptosis (Toyokuni et al., 2017). Thus, iron chelators (e.g., deferoxamine) and several lipophilic antioxidants (e.g., α-tocopherol) can rescue ferroptosis (Yagoda et al., 2007; Yang and Stockwell, 2008; Zilka et al., 2017). Additionally, ROS produced through the Fenton reaction catalyzed by iron contributes to the initiation of ferroptosis (Toyokuni et al., 2017).

## Accumulation of Lipid Peroxides

Under physiological condition, lipid peroxides (e.g., PE-OOH) are reduced to its corresponding lipid alcohols (e.g., PE-OH) by reductase to protect cells against oxidative stress (Brigelius-Flohe and Maiorino, 2013; Yang et al., 2014). Here, we roughly divide the processes that cause the accumulation of lipid peroxides into two aspects: processes that facilitate the formation of lipid peroxides and processes that inhibit the reduction of lipid peroxides.

### Processes That Inhibit the Reduction of Lipid Peroxides

Toxic lipid peroxides are reduced to nontoxic lipid alcohols by GPX4 in the presence of glutathione (GSH), a cofactor of GPX4 (Brigelius-Flohe and Maiorino, 2013; Yang et al., 2014). GPX4 prevents cells against ferroptosis by eliminating intracellular lipid ROS and the inhibition of GPX4 triggers ferroptosis (Yang et al., 2014, 2016; Kinowaki et al., 2018). Containing eight nucleophilic amino acids (i.e., one selenocysteine and seven cysteines), GPX4 can react with electrophiles in the cell (Yang et al., 2016). Selenium is required for GPX4 to maintain its ferroptosis-resistance activity and replacing selenocysteine with cysteine sensitizes cells to ferroptosis (Friedmann Angeli and Conrad, 2018; Ingold et al., 2018). The inactivation or absence of GPX4 causes the accumulation of lipid peroxides, which is regarded as the lethal signal of ferroptotic cell death (Yang et al., 2016; Kagan et al., 2017). Thus, the inhibition of GPX4 is the critical step in ferroptosis. Several pathways are already known to lead to the inhibition of GPX4 and we review them here in association with their corresponding inducers, four small-molecule compounds (i.e., erastin, RSL3, FIN56, and FINO_2_) (Dolma et al., 2003; Yang and Stockwell, 2008; Shimada et al., 2016b; Gaschler et al., 2018a) (Table 1).

**Table 1:** Inducers that inhibit GPX4.

Inducers	Drugs	Mechanisms	Reference
Erastin	Sorafenib (an anti-cancer drug)	Depletion of GSH	Dixon et al., 2012;
Erastin derivatives	sulfasalazine (an anti-inflammatory drug)	Inactivation of GPX4	Friedmann Angeli et al., 2014; Lachaier et al., 2014;
Glutamate	Artesunate(an anti-malaria drug)		Eling et al., 2015; Roh et al., 2017
BSO			
RSL3	Altretamine (an anti-cancer drug)	Directly bind to GPX4 Inactivate GPX4	Woo et al., 2015; Yang et al., 2016
FIN56		Promote the degradation of GPX4	Shimada et al., 2016b
		Decrease the abundance of GPX4	
FINO_2_		Indirectly inactivate GPX4 Directly oxidize iron and lipid	Abrams et al., 2016; Gaschler et al., 2018a

#### Erastin

Erastin inhibits system x_c_^_^, a Na^+^-independent cystine/glutamate antiporter that can import a single molecule of cystine into the cells and export glutamic acid out of the cells in an ATP-dependent manner (Choi, 1988; Murphy et al., 1989; Dixon et al., 2012). System x_c_^_^ is a heterodimer composed of SLC7A11 and SLC3A2, linked by disulfide (Sato et al., 1999). How erastin inhibits system x_c_^_^ is unknown, but a change in SLC7A11 may be responsible for this inhibition (Dixon et al., 2012). The inhibition of system x_c_^_^ decreases the uptake of cystine, the oxidized form of cysteine. In cells, GSH synthase and glutamate cysteine synthase synthesize GSH with glutamate, glycine, and cysteine, which is reduced from cystine in the cell as substrates (Kagan et al., 2017). The decrease of cystine leads to the decrease of cysteine and the depletion of GSH. Serving as a cofactor of GPX4, GSH is required for the ferroptosis-resistance activity of GPX4. Inhibition of GPX4 suppresses the reduction of lipid peroxides to lipid alcohols, which leads to the accumulation of lipid peroxides. In addition, as one of the most abundant antioxidants, GSH functions to protect cells against ROS in the cell (Toyokuni et al., 2017). So, the depletion of GSH leads to the disequilibrium of antioxidant defense and the increase of lipid ROS. Thus, erastin induces ferroptosis by inhibiting the synthesis of GSH via decreasing the uptake of cysteine. The inhibition of GSH inactivates the GPX4 and reduces the oxidation resistance of the cell. These two processes lead to the accumulation of lipid peroxides and lipid ROS. Both of them are harmful to the cells by damaging the intracellular organics (e.g., proteins, lipids, and nucleic acids). L-buthionine sulfoximine (BSO; Friedmann Angeli et al., 2014), sorafenib (Lachaier et al., 2014), and artesunate (Eling et al., 2015) also induce ferroptosis by the depletion of GSH. Consistent with the above findings, reagents or processes that increase the intracellular abundance of cystine/cysteine can rescue erastin-induced ferroptosis, such as β-mercaptoethanol (β-ME), transsulfuration, and the processes that enhance the synthesis of cysteine (Ishii et al., 1981; Hayano et al., 2016; Shimada and Stockwell, 2016).

#### RSL3

RAS-selective lethal 3, a compound containing an electrophilic moiety and a chloroacetamide moiety, can react with the nucleophilic moiety of GPX4. RSL3 can react with the eight nucleophilic amino acid residues of GPX4, but the binding between RSL3 and GPX4 is mainly driven by the reaction with selenocysteine, the nucleophilic amino acid residue at the active site of GPX4. This binding directly leads to the inactivation of GPX4 (Yang et al., 2016). Similar with RSL3, altretamine, an anti-cancer drug that was approved by FDA, was newly identified as a direct inhibitor of GPX4. However, the mechanism of the GPX4-resistance activity of altretamine remains unknown (Woo et al., 2015).

#### FIN56

Shimada and Stockwell (2016) screened a compound that induced cell death while lacking the activation of caspases 3 and 7. The compound was named as CIL56 which was then identified as an inducer of ferroptosis for its killing activity on oncogenic RAS cells. However, further studies demonstrated that antioxidants and iron chelators only rescued the lethal effect of CIL56 at its low concentrations. The authors guessed there must be other forms of cell death in the cell during a high concentration of CIL56. Subsequently, they found an analog of CIL56 named FIN56 (ferroptosis inducing 56) that retained oncogenic RAS selectivity while lacking the ability to trigger other forms of cell death. FIN56 induces ferroptosis by two different pathways: promoting the degradation of GPX4 and reducing the abundance of CoQ_10_ (i.e., an antioxidant in the cell). How FIN56 promotes the degradation of GPX4 is unknown, but the enzymatic activity of acetyl-CoA carboxylase (ACC) is required for this pathway. FIN56-mediated mevalonate pathway reduces the abundance of CoQ_10_ and we will describe this pathway below (Shimada et al., 2016b).

#### FINO_2_

FINO_2_ is an endoperoxide-containing 1,2-dioxolane that requires both an endoperoxide moiety and a nearby hydroxyl head group for its ferroptosis-inducing ability (Abrams et al., 2016; Gaschler et al., 2018a). Unlike previous ferroptosis inducers, FINO_2_ neither influences the metabolism of amino acids (i.e., as erastin does) nor binds directly to GPX4, leading to its inactivation (i.e., as RSL3 does). FINO_2_ does not decrease the abundance of GPX4 (i.e., as FIN56 does), but the activity of GPX4 is subdued in the cells treated with FINO_2_. β-ME can react with cystine to form a disulfide outside the cell (Ishii et al., 1981). Importation of this mixed disulfide into the cell through neutral amino acid transporters bypasses system x_c_^_^ and elevates intracellular cysteine availability to prevent ferroptosis. Consistent with this mechanism, β-ME fully rescues erastin-induced ferroptosis in the cell. However, RSL3-induced ferroptosis cannot be rescued by β-ME because RSL-3 inactivates GPX4 by direct binding to GPX4 without changing the metabolism of amino acids. Interestingly, β-ME partially rescues FIN56-induced ferroptosis. This may be attributed to the reason that the increase of intracellular cysteine promotes the biosynthesis of GSH, a cofactor of GPX4. In the cells treated with FIN56, the abundance of GPX4 is decreasing while the activity of the remaining GPX4 is increasing via the elevation of GSH. Similar to FIN56, FINO_2_-induced ferroptosis can also be partially rescued by β-ME. Although the mechanism was unknown, researchers hypothesized that FINO_2_ indirectly inhibited the activity of GPX4. Meanwhile, FINO_2_ was found to oxidize ferrous iron directly. Contained in FINO_2_, 1,2-dioxolanes can react with ferrous iron to produce oxygen-centered radicals, as in Fenton chemistry. Besides, FINO_2_ can also oxidize lipids, providing another source of lipid peroxides (Abrams et al., 2016; Gaschler et al., 2018a).

### Processes That Facilitate the Formation of Lipid Peroxides

Lipids play crucial roles in the energy supply and the composition of intracellular membrane system. The oxygenation of phospholipid (PL) (e.g., PE, phosphatidylcholine, cardiolipin) facilitates ferroptosis in the cells (Yang et al., 2016; Kagan et al., 2017; Shah et al., 2018). Lipid peroxides are known to be produced by three major pathways in the cells, all of which require iron: (1) lipid ROS produced through the Fenton reaction by iron in a non-enzymatic manner; (2) lipid peroxides generated by oxygenation and esterification of polyunsaturated fatty acids (PUFAs; Yang et al., 2016; Doll et al., 2017; Kagan et al., 2017; Shintoku et al., 2017); and (3) lipid peroxides produced by lipid autoxidation in an iron-catalyzed manner (Soupene et al., 2008; Table 2). The Fenton reaction is an inorganic chemical reaction and widely found in nature; we mainly discuss the two intracellular organic reaction. Both of these two pathways are certainly involved in ferroptosis, while which one is the major contributor or whether these two pathways are paralleled is controversial. More evidence were in favor of conclusion that peroxidation of PUFAs was the major regulator of ferroptosis.

**Table 2:** Pathways that produce lipid ROS.

Substrates	Reactions	Enzymes and processes	ROS	Inhibitors	Reference
AA and AdA	Lipid peroxidation	LOXs, ACSL4, LPCAT3	AA-OOH-PE	Iron chelators	Yang et al., 2016; Doll et al., 2017; Kagan et al., 2017; Shintoku et al., 2017
		Esterification and peroxidation	AdA-OOH-PE	Lipophilic reductants	
Long-chain PUFAs	Lipid autoxidation	Autoxidation	L-OOH	RTAs	Ingold and Pratt, 2014; Shah et al., 2018
Lipid	Fenton reaction	Fe^2+^ + H_2_O_2_ = Fe^3+^ + •OH + HO-	•OH	Unknown	Pignatello et al., 2006; Stadtman, 1993

#### Oxygenation and Esterification of PUFAs

In 2016, Kagan et al. screened 350 species of PLs and identified oxidized AA-containing PE (AA-PE) as a ferroptotic cell death signal. AA is a type of PUFAs that can be elongated into adrenoyl (AdA) by elongase (Kagan et al., 2017). The accumulation of the oxygenated AA-PE and AdA-PE induces ferroptosis in the cells. A further study found that exogenous AA and AA-OOH-PE enhanced ferroptosis, while AA-OOH did not. This result indicated that it was AA-OOH-PE rather than other types of PL-OOH that induced ferroptosis. The formation of AA-OOH-PE from AA in the cells requires three enzymes: lipoxygenases (LOXs), acyl-CoA synthetase long-chain family 4 (ACSL4), and lysophosphatidylcholine acyltransferase 3 (LPCAT3; Dixon et al., 2015; Yang et al., 2016; Doll et al., 2017; Kagan et al., 2017; Shintoku et al., 2017). This process includes the ACSL4-catalyzed formation of AA-CoA followed by the LPCAT3-controlled esterification of AA-CoA into AA-PE and the end process, oxidation of AA-PE to AA-OOH-PE by LOXs. After the formation of AA-CoA, there are two alternative sequences to form AA-OOH-PE: oxidation followed by esterification of AA-CoA or the opposite order. When the level of AA-OOH-PE overwhelms the threshold of the cell, ferroptosis occurs.

The ACSL family consists of proteins that are mainly expressed on the endoplasmic reticulum and mitochondrial outer membrane. ACSLs are responsible for the formation of acyl-CoAs from fatty acids. There are five isoforms of ACSLs, ACSL1, ACSL3, ACSL4, ACSL5, and ACSL6 (Soupene et al., 2008), where only ACSL4 has a high correlation with ferroptosis. Knockout of *gpx4* led to the ferroptotic cell death, while *gpx4* and *acsl4* double-KO cells could survive and proliferate normally. This conclusion indicated that ACSL4 was required for ferroptosis in the absence or inactivation of GPX4 (Doll et al., 2017). Additionally, the expression of ACSL4 in ferroptosis-resistant cells (e.g., K562) is distinctly lower than that in ferroptosis-sensitive cell lines (e.g., HL60). Therefore, ACSL4 can be regarded as a marker of ferroptosis sensitivity (Yuan et al., 2016). It should be emphasized that ACSL4 is not the only enzyme that can activate AA and AdA. Other ACSLs can also activate AA (e.g., ACSL3), but a high concentration of AA and AdA are required for this effect. Normally, the abundance of AA is lower than that of other fatty acids and ACSL4 preferentially activates AA and AdA for the synthesis of PLs (Doll et al., 2017). Thus, ACSL4 is the major regulator of AA.

#### Lipid Autoxidation Catalyzed by Iron

Lipoxygenases are non-heme-iron-containing dioxygenases that catalyze the insertion of oxygen into PUFAs at the bis-allylic position in non-bilayer PL arrangements (Kuhn et al., 2005; Kagan et al., 2017). Several enzymes can oxidize AA in the cells (Massey and Nicolaou, 2011), but only inhibitors of LOXs can prevent ferroptosis (Kagan et al., 2017). It seems that ferroptosis is a LOXs-dependent process, while Ron Shah found that not all inhibitors of LOX rescue ferroptosis. The compounds that could inhibit ferroptosis were all identified as radical-trapping antioxidants (RTAs) whose function is to protect cells against autoxidation, an autocatalytic classic free radical chain reaction that can generate lipid hydroperoxides in the presence of iron (Shah et al., 2018). This report stated that lipid autoxidation might be the final process of ferroptosis rather than the LOXs-controlled lipid peroxidation.

While this conclusion raises another question: AA-OOH-PE is identified as the cell death signal of ferroptosis (Kagan et al., 2017), but most end-products of lipid autoxidation are lipid hydroperoxides which contain different kinds of lipid peroxides; can other forms of hydroperoxides, such as LOOH, induce ferroptosis? The answer is definite. We overemphasize AA and AdA because they show greater changes and higher relevance than other long-chain PUFAs. Other long-chain PUFAs can also sensitize cells to ferroptosis when the total concentration of LOOH reaches the threshold in a given cell type (Shah et al., 2018). A feasible assumption was given in this study: the activation of three enzymes (i.e., ACSL4, LPCAT3, and LOX) increases the levels of intracellular LOOH, which contributes to ferroptosis just at the initiation stage. Once ferroptosis is initiated, lipid autoxidation leads to the final cell death process. LOOH and the availability of low-valent metals (e.g., Fe^2+^) increase the initiation rate of autoxidation (Shah et al., 2018). RTAs protect cells against autoxidation (Ingold and Pratt, 2014) and when the initiation rate of autoxidation overwhelms the intrinsic RTA capacity of a given cell, lipid autoxidation occurs.

In brief, this study put forward three proposals: (1) lipid autoxidation is certainly involved in ferroptosis; (2) lipid autoxidation might be the final process of ferroptosis rather than the LOXs-controlled lipid peroxidation; and (3) lipid autoxidation led to the end cell death process, while lipid peroxidation just contributes to the initiation of lipid autoxidation. In addition, the unsaturation of PUFAs makes the molecule more sensitive to autoxidation (Shah et al., 2018). This explains why PUFAs are the chief substrates to be oxidized in ferroptosis.

It is already known that the oxidation of PUFAs is harmful to the cells under the depletion of GSH; however, it is interesting to find that this lethal effect is negligible at normal levels of intracellular GSH (Yang et al., 2016). Thus, we conclude that the loss of GSH, depletion or inactivation, may contribute more to ferroptosis than the oxidation of PUFAs; that is, the processes that inhibit the reduction of lipid peroxides contribute greater on the initiation of ferroptosis than the processes that facilitate the formation of lipid peroxides. Thus, we conclude that ferroptosis is an oxidized cell death caused by a decrease of reduction reactions. As could be expected, different RTA capacities and different LOXs levels influence the ferroptosis-sensibility of the cell. The diversities of the basal RTA and LOXs in the cells depend on the cell types, physiological conditions, and even individual life styles. This assumption further confirms the point that ferroptosis is a physiological process which might present in plenty of cell types or all types. Maybe ferroptosis does not happen on a given cell just because the lipid peroxides haven’t reached its threshold.

## Iron and Ferroptosis

As one of the most abundant transition metals, iron is an essential element for nearly all organisms. The total iron in the adult human is ∼3–5 g and up to 80% of which is found in hemoglobin; less than 20% is stored in macrophages and hepatocytes. In human, recycling iron released by macrophages from aged red blood cells satisfies more than 90% iron demand and only 1 mg of iron per day is absorbed from a diet as “new iron.” There does not exist a physiological mechanism of iron loss and iron loss mainly through desquamation of epithelial cells in the intestine and the skin, and through bleeding (e.g., menstruation and childbirth). In human, the deficiency of iron causes anemia which affects millions of people worldwide and excess iron leads to an inherited disease hemochromatosis (Muckenthaler et al., 2008; Lawen and Lane, 2013). There are two forms of iron in the cells: Fe(II) and Fe(III). On account of Fe(II)’s ability of transfer electrons and high solubility, Fe(II)-containing proteins always serve as cofactors and catalysts participating in various oxidation–reduction reactions, whereas iron is stored and transported in its stable Fe(III) form. However, the ease in electrons transfer also makes iron poisonous to cells for excess iron atoms can donate electrons to O_2_ and H_2_O_2_ to generate superoxide anion and the hydroxyl radical, both of which can damage cells by oxidizing proteins, lipids, and nucleic acids. Moreover, the mixture of Fe(II) and H_2_O_2_ can oxidize organics (i.e., alcohol, ester) to generate ROS by the Fenton reaction (Pignatello et al., 2006). However, studies demonstrate that a high level of ROS accumulates in massive tumor cells (Kasai, 1997) and excess iron is regarded as a risk factor of tumorigenesis (Toyokuni, 2002). Both the conclusions seem to state the same truth, that excess ROS generated by iron might promote the development of a tumor. Nevertheless, the contradiction lies in the evidence that iron-dependent accumulation of ROS induces ferroptosis, a process that inhibits tumor cells. One hypothesis was put forward to account for the contradiction that in cancer cells, iron and thiol redox signaling maintain a balance to help cells escape ferroptosis (Toyokuni et al., 2017), but this viewpoint needs more proof.

### Accessibility of Iron

#### Iron Uptake

Two mechanisms are responsible for the transport of non-heme iron into cells: transferrin (Tf)-dependent manner and Tf-independent manner. Tf is a glycoprotein which has two high-affinity sites for Fe(III). Normally, Tf is about 30% binding with iron and almost all iron is transported into cells by Tf-dependent manner. However, when the binding between Tf and iron is saturated, iron can be transported into cells in a Tf-independent manner. The ferric iron is reduced to ferrous iron in the presence of membrane-bound ferrireductases. The ferrous iron is then transported into cells by divalent metal transporter 1 (DMT1; Richardson and Ponka, 1997; Hentze et al., 2010). Non-heme ferric iron absorbed by intestinal epithelial cells is just in this manner while the pathway about the absorption of heme from diet (e.g., meat) by intestinal epithelial cells is still unclear. Recent studies indicated that two candidates, heme carrier protein 1 (HCP1) and heme responsive gene-1, might be involved in the uptake of heme in intestinal (Shayeghi et al., 2005; Rajagopal et al., 2008). Under a physiological condition, Tf can bind two Fe(III) to form diferric Tf, which is then bound to the high-affinity Tf receptor 1 (TfR1) on the surface of cells. The Tf-Fe_2_-TfR1 complex is transported into cells by endocytosis to form endosomes. The endosomes release iron from the complex in the acidic environment of the endosomes. Free ferric iron is then reduced to ferrous iron, which is subsequently transported into cytoplasm by DMT1. The ferrous iron becomes part of labile iron pool (LIP), while the endosomes containing Apo-Tf-TfR1 complex return to the surface of the cells, waiting for the release of Apo-Tf and preparing for the next recycling (Richardson and Ponka, 1997; Hentze et al., 2010). The iron in the cells is then stored in ferritin, exported out of the cells by ferroportin (FPN), or utilized for the synthesis of proteins.

#### Iron Utilization, Export, and Store

In the cytoplasm, most of iron is transported into mitochondria for the synthesis of heme and Fe–S clusters, whereas a small portion of iron is used for the formation of iron-containing proteins. FPN is the sole known intracellular exporter that can transport iron out of the cells, while the mechanism of FPN-mediated iron export remains unclear. On account of the evidence that the export of iron requires an extracellular ferroxidase activity, scientists speculate that FPN export iron in its ferrous form (Donovan et al., 2000). The ferrous iron transported out of cells is oxidized to ferric iron by extracellular ferroxidase. The free ferric iron is then bound to Tf in the circulation and the Tf-Fe_2_ complex is transported to other cells. Iron that is not used or exported is stored in ferritin. Ferritin is a heteropolymer formed with 24 subunits of ferritin heavy chain 1 (FTH1) and ferritin light chains (FTL). FTH is in charge of the hold of iron atoms and FTL may be involved in the transfer of electrons. Each of FTH1 can accommodate 4500 ferrous iron atoms, which are then oxidized to ferric iron by FTH1 in an oxygen-dependent manner (Arosio and Levi, 2010). The release of iron from ferritin is controlled under physiological condition (Kurz et al., 2008). Recent studies demonstrated that the nuclear receptor coactivator 4 (NCOA4)-mediated ferritinophagy played a vital role in the release of iron from ferritin. NCOA4 binds to ferritin and delivers it to lysosomes for degradation (Mancias et al., 2014). The degradation releases iron and increases the abundance of iron in the cell. Thus, several research indicated that NCOA4-mediated ferritinophagy promoted ferroptosis by increasing the availability of intracellular iron (Gao et al., 2016; Hou et al., 2016; Masaldan et al., 2018; Zhang et al., 2018).

### Regulation of Iron Homeostasis

#### Systemic Iron Regulation

Iron regulation contains two levels: systemic and cellular levels. FPN serves as an important transporter in the systemic iron regulation. Systemic iron is sensed by liver which can secrete hormone hepcidin, a peptide that negatively regulates systemic iron. Thus, systemic iron regulation is controlled by the hepcidin-dependent manner and the hepcidin-independent manner. When the plasma iron level meets the systemic iron demand, the liver increases the secretion of hepcidin into blood. The hepcidin binds to FPN and changes the structure of FPN, followed by its phosphorylation. Phosphorylated FPN is subsequently internalized and ubiquitinated. Ubiquitinated FPN is degraded in the lysosomes. FPN can also regulate iron by a hepcidin-independent manner. When the intracellular iron level is decreased, FPN goes through a lack of binding with iron and causes a conformational change of FPN. The conformational change makes it easy for FPN to be ubiquitinated and the ubiquitinated FPN is subsequently internalized and degraded in the lysosome (Sangkhae and Nemeth, 2017). These two manners both decrease the abundance of FPN and lead to a decrease in iron export.

#### Cellular Iron Regulation

Cellular iron homeostasis depends on the iron regulatory protein 1, 2 (IRP1, IRP2) and iron responsive elements (IREs) system. IRPs are proteins that can bind to the 5′ or 3′ untranslated regions (UTRs) of IRE’s mRNAs. These key mRNAs are involved in the iron regulation, including that of iron uptake (e.g., DMT1, TfR1), iron sequestration (e.g., subunits of ferritin: FTH1, FTL), and iron export (e.g., FPN). When the iron is insufficient in the cell, IRPs bind to 5′ IREs of ferritin and FPN to inhibit their translation and to 3′ IREs in TfR1 to suppress its degradation. When the iron satisfies the demand, the IRPs is degraded and these bindings stop (Anderson et al., 2012; Thompson and Bruick, 2012). It is interesting to find that the iron homeostasis in both manners is regulated by iron. In systemic iron regulation, the level of iron is sensed by liver and liver secretes hormone hepcidin according to iron abundance. In cellular iron level, the loss of IRP1-IREs binding activity depends on the insertion of 4Fe–4S cluster. As for the IRP2, a newly discovered FBXL5-dependent E3 ligase complex catalyzes the ubiquitination and proteasomal degradation of IRP2, while keeping the stability of FBXL5 requires iron and oxygen (Salahudeen et al., 2009; Vashisht et al., 2009; Anderson et al., 2012; Thompson and Bruick, 2012).

### Iron and Ferroptosis

**FIGURE 1:**
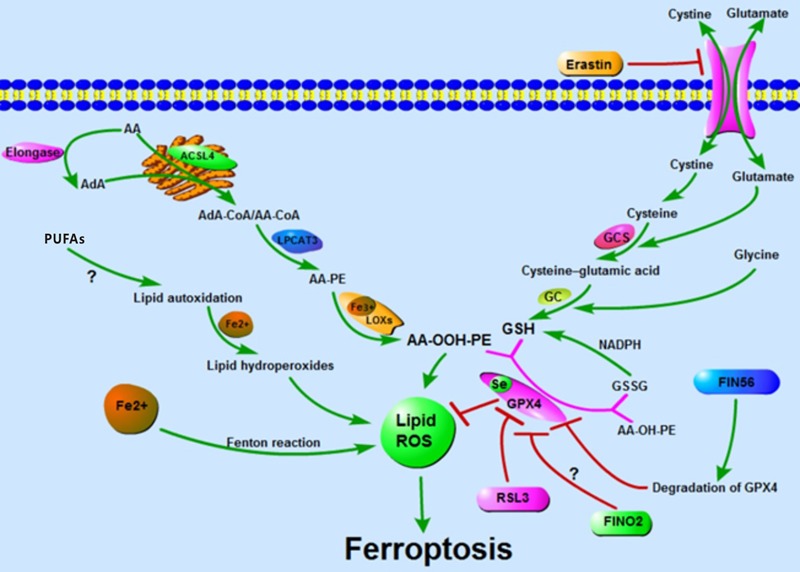
Accumulation of lipid ROS: oxidation of lipid, lipid autoxidation, and Fenton reaction facilitate the generation of lipid ROS. The metabolism of amino acids suppresses the synthesis of GSH and the activity of GPX4, thus inhibiting the reduction of lipid ROS. The accumulation of lipid ROS leads to ferroptosis.

Ferroptosis is named for the reason that the process is iron-dependent and can be prevented by iron chelators. The alteration in the transcription of iron regulation genes (e.g., *IREB2, FBXL5, TFRC, FTH1*, and *FTL*) affects the sensibility of erastin-induced ferroptosis and this sensibility is positively correlated with the abundance of intracellular iron. Similarly, in the ferroptosis-sensitive cells, the Tf is increasing and the FPN is reducing (Gao et al., 2015; Ma et al., 2016). The lysosomes contain a high concentration of iron and their disorder also contributes to ferroptosis (Ma et al., 2016). Furthermore, the extracellular iron level sensitizes cells to ferroptosis *in vivo* and *in vitro*: high-iron diets trigger ferroptosis in mice and adding iron to the extracellular matrix sensitizes cells to the ferroptotic cell death (Wang et al., 2017). Others, such as heat shock protein family B member 1 (HSPB1), inhibit ferroptosis by reducing intracellular iron levels and by upholding GSH in its reduced form. HSPB1 inhibits the TfR1-mediated iron uptake via stabilization of the cortical actin cytoskeleton. This process inhibits the endocytosis and the recycling of Tf, which reduces the level of intracellular iron (Arrigo et al., 2005; Chen et al., 2006; Sun et al., 2015). In addition, heme oxygenase 1 (HO-1) and phosphorylase kinase catalytic subunit gamma 2 (PHKG2) mediate ferroptosis by regulating the abundance of iron (Kwon et al., 2015; Yang et al., 2016). Taking all research into account, all reports stressed the importance of intracellular-free iron in the ferroptosis. Even the proteins in these reports are those involved in the iron uptake, utilization, store, export, or the regulator of iron and all of them regulate ferroptosis by mediating the intracellular iron. To date, there are three known pathways participating in the iron-dependent accumulation of lipid ROS in ferroptosis: (I) ROS generated via the Fenton reaction by iron, an inorganic chemical reaction in a non-enzymatic manner; (II) ROS produced by lipid autoxidation which is controlled in an iron-catalyzed enzymatic manner; and (III) ROS produced from the oxidization of AA by iron-containing LOXs. Although the vital role of iron in ferroptosis is confirmed, how iron regulates ferroptosis is still unknown. Much more research is required to illuminate the relationships between iron and ferroptosis (Figure 1).

## Other Pathways of Ferroptosis

### Mevalonate Pathway

The toxic small-molecule FIN56 is required for mevalonate pathway-mediated ferroptosis. FIN56 can activate its own target protein SQS besides inducing ferroptosis by decreasing the abundance of GPX4. SQS links two molecules of farnesyl pyrophosphate (FPP) to form one molecule of squalene. The previous studies had demonstrated that idebenone, a metabolite of FPP, rescued FIN56-induced ferroptosis (Tansey and Shechter, 2000; Shimada et al., 2016b). The activation of SQS by FIN56 leads to a decrease in FPP and idebenone. Furthermore, idebenone is a hydrophilic analog of CoQ_10_ which is a powerful antioxidant in the cell (Gueven et al., 2015). In sum, FIN56 reduces the level of idebenone by activating SQS in the cells, which results in a decreased anti-oxidation activity in the cells.

### P53 and Its Dual Effects on Ferroptosis

P53 is a famous tumor suppressor that has been studied for decades. The abilities of p53 to mediate cell-cycle arrest, senescence, and apoptosis are widely believed to be responsible for its tumor suppression function (Green and Kroemer, 2009; Bieging et al., 2014; Khoo et al., 2014). P53 suppresses tumors by serving as a DNA-binding transcription factor that influences the expression of its target genes. In 2012, Gu et al. found that the mutation of three normally acetylated lysine residues (3KR[K117R+K161R+K162R]) in the DNA-binding domain of p53 led to a deficiency in acetylation, referred to as p53^3KR^. Notably, the mutant p53^3KR^ model which was defective for the three conventional functions of p53 could also suppress tumor growth (Li et al., 2012). Therefore, there must be an additional pathway that mediates tumor development. P53^3KR^ was then found to target the gene *SLC7A11* and the binding led to the decrease of SLC7A11, which then sensitizes cells to ferroptosis (Jiang et al., 2015). P53^3KR^ inhibited tumor growth by inducing ferroptosis while p53^4KR98^, a mutant containing four mutations of acetylated lysine residues (K98R+3KR), lost the tumor suppression activity (Wang et al., 2016). Therefore, the acetylation of K98 is crucial to p53-mediated ferroptosis. However, there is no straight evidence indicating that wild-type p53 can suppress tumor growth by inducing ferroptosis. Maybe p53^3KR^ gains a ferroptosis-inducing capacity while p53^4KR^ loses that.

Setting this question aside, p53-mediated ferroptosis has varying effects. A study published in 2017 showed that p53 suppresses ferroptosis in cancer cells. Cells treated with nutlin-3, which is used to stabilize p53, show a delayed onset of ferroptosis in the presence of p21, a transcriptional target gene of p53 encoded by gene *CDKN1A*. In other words, ferroptosis is inhibited in response to nutlin-3 treatment. Cells treated with nutlin-3 show decreased activity of system x_c_^_^ but increased GSH for the inhibited degradation of GSH (Tarangelo et al., 2018). The inhibition of system x_c_^_^ facilitates ferroptosis, while in this report, the inhibition of system x_c_^_^ and the suppression of ferroptosis occur simultaneously. There must exist a pathway that counteracts the effect of the inhibition of system x_c_^_^. Whether the increase in GSH is responsible for the neutralization of inhibition of system x_c_^_^ is unclear. Meanwhile, another study found that p53 suppresses ferroptosis in human colorectal cancer (CRC), while in other cancer cells, p53 acts as a positive regulator of ferroptosis. P53 inhibits ferroptosis by transforming dipeptidyl-peptidase-4 (DPP4) to the nucleus from the membrane to form the DPP4-p53 complex in CRC. Moreover, p53 promotes the expression of SLC7A11 in CRC cells, while in other tumor cells (e.g., U2OS and MCF7 cells), p53 inhibits its expression. Is the converse effect on p53-mediated ferroptosis due to different cell types? (Xie et al., 2017).

**FIGURE 2:**
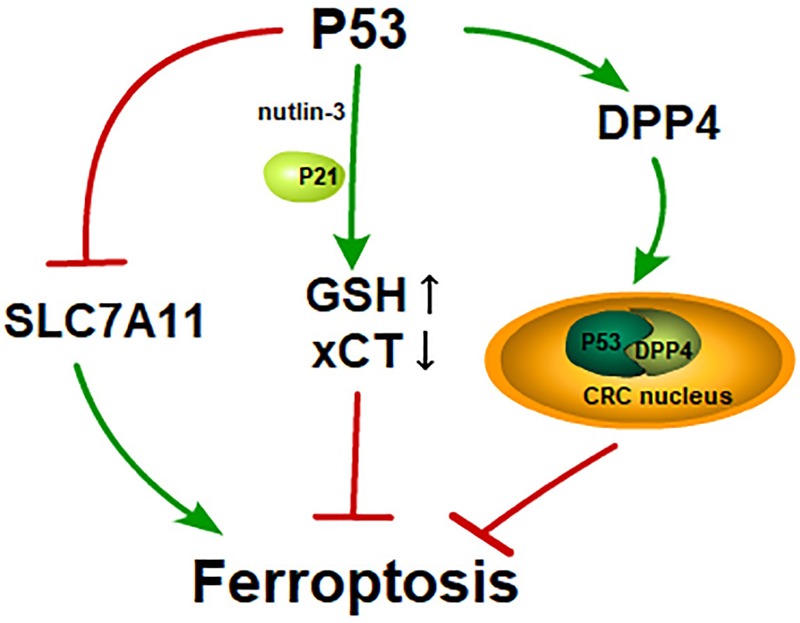
P53 and its dual effects on ferroptosis: P53 induces ferroptosis by inhibiting SLC7A11 like erastin; GSH is increased and xCT is inhibited in the cells treated with nutlin-3, a reagent that is used to stabilize p53. Cells treated with nutlin-3 show a delayed onset of ferroptosis in the presence of p21; In CRC, p53 activates DPP4 and transfers DPP4 to CRC nucleus from cytoplasm. The p53-DPP4 compound inhibits ferroptosis.

In short, p53 performs dual regulatory functions in ferroptosis. Perhaps we have not yet found the key regulator of p53-mediated ferroptosis or perhaps p53-mediated ferroptosis shows different effects under different conditions (Figure 2).

### Uncertainty Regarding p62 and NRF2 in Ferroptosis

**FIGURE 3:**
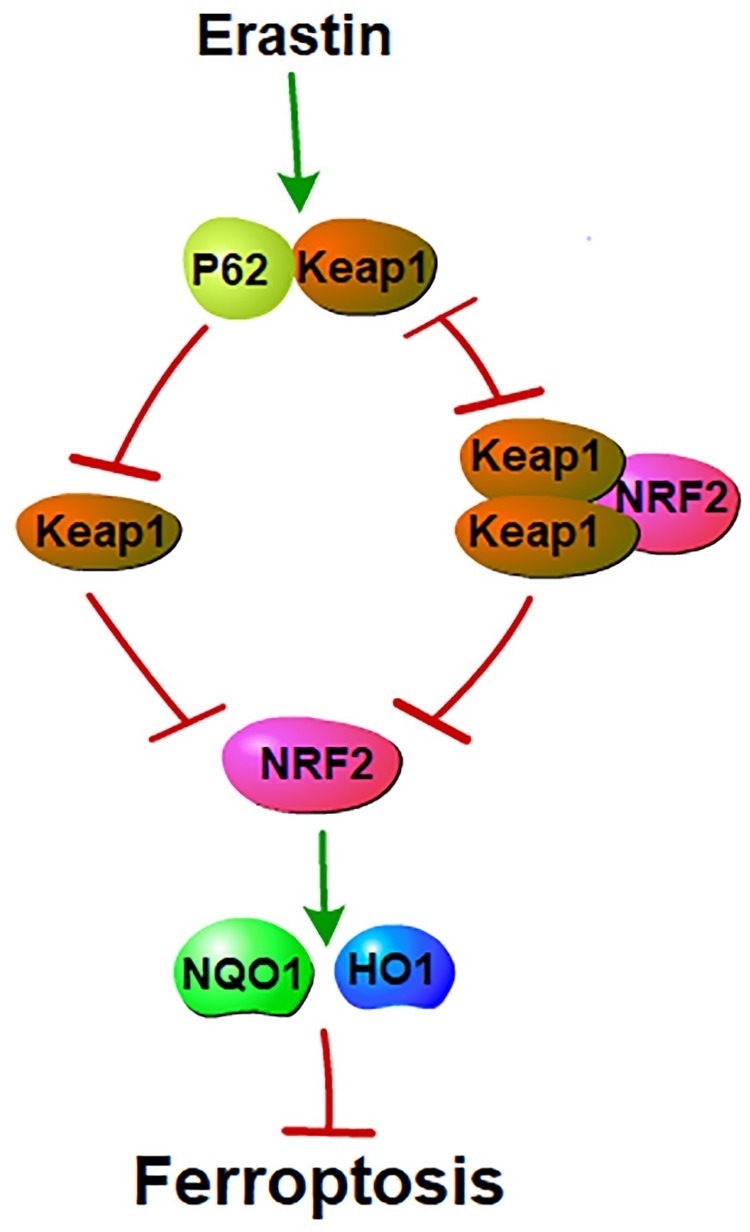
P62 and NRF2 in ferroptosis: NRF2 and p62 binds competitively to Keap1. Ferroptosis inducers facilitate the interaction between p62 and Keap1. This interaction inhibits Keap1. Inhibition of Keap1 prevents the binding between Keap1 and NRF2. Interaction of Keap1 and NRF2 triggers the degradation of NRF2. NRF2 mediated ferroptosis by regulating genes that involve in the metabolisms of iron and ROS. Thus, p62-Keap1-NRF2 pathway negatively mediated ferroptosis.

In 2016, Sun showed that the nuclear factor erythroid 2-related factor 2 (NRF2) negatively regulates ferroptosis in a linear relationship referred to as the p62-keap1-NRF2 pathway. NRF2 and p62 bind competitively to Keap1 (Komatsu et al., 2010). Two molecules of Keap1 interact with one NRF2 molecule and this interaction facilitates the ubiquitylation and degradation of NRF2 (Padmanabhan et al., 2006; Tong et al., 2007). NRF2 inhibits ferroptosis by increasing the expression of target genes involved in the metabolism of iron and ROS, such as quinone oxidoreductase 1 (NQO1) and HO1. Furthermore, a high NRF2 expression is related to a poorer overall survival rate in patients with glioma and the activation of the NRF2-Keap1 pathway promotes system x_c_^_^ (i.e., NRF2 inhibits ferroptosis; Fan et al., 2017). However, another study reached the opposite conclusion. In HCC, ferroptosis inducers facilitated the expression of NRF2 (i.e., NRF2 promotes ferroptosis; Sun et al., 2016). These two phenomena are paradoxical in terms of NRF2-mediated ferroptosis. Is there a feedback loop between NRF2 and ferroptosis? Or does NRF2-mediated ferroptosis show different effects on different cell types (Figure 3)?

## New Insight of Ferroptosis

### Mutant RAS Is Dispensable for Ferroptosis

Erastin was initially found to selectively induce ferroptosis in RAS mutant tumor cells, so mutant RAS was identified as an indispensable element of ferroptosis. Previous reports showed that the inhibition of MEK by U0126 fully rescued ferroptosis, and the authors concluded that the RAS-ERK pathway was responsible for the lethality of erastin (Yagoda et al., 2007). However, further studies found that another more selective and potent MEK1/2 inhibitor, PD0325901, failed to suppress ferroptosis (Gao et al., 2015). The mutant RAS seems to be unnecessary for ferroptosis. Consistent with the above findings, further studies have found that ferroptosis occurs in normal RAS cells (Friedmann Angeli et al., 2014; Chen et al., 2015; Eling et al., 2015; Matsushita et al., 2015; Yu et al., 2015). Moreover, cells overexpressing mutant RAS have shown resistance to erastin-induced ferroptosis (Schott et al., 2015). Thus, we can conclude that mutant RAS is dispensable for ferroptosis (Yuan et al., 2016).

### Biomarkers of Ferroptosis

Lipid peroxides, increased levels of PTGS2, and the decrease of nicotinamide adenine dinucleotide phosphate (NADPH) can be recognized as biomarkers of ferroptosis (Yang et al., 2014, 2016; Shimada et al., 2016a). Levels of malondialdehyde (MDA), an end-product of lipid peroxides, can replace lipid peroxides as a biomarker. *PTGS2*, a gene encoding cyclooxygenase-2 (COX-2), markedly increased in cells treated with ferroptosis inducers and this increase is not affected by the inhibitors of PTGS. These findings suggested that *PTGS2* did not regulate ferroptosis and the increase in *PTGS2* was a suitable marker for ferroptosis (Yang et al., 2014). Normally, GPX4 protects cells against ferroptosis by catalyzing GSH and toxic PE-AA-OOH into oxidized GSH (GSSG) and nontoxic PE-AA-OH. GSSG is then converted into GSH by GSH reductase (GR) in the presence of NADPH. Therefore, NADPH, a coenzyme of GR, plays a vital role in maintaining the abundance of intracellular GSH. Furthermore, basal NADPH abundance of a given cell has been shown to correlate negatively with ferroptosis sensitivity (Shimada et al., 2016a). Moreover, NADPH may link ferroptosis and necroptosis, which we describe below (Tonnus and Linkermann, 2016).

### Relationships Between Ferroptosis and Other Forms of Cell Death

The cells in ferroptosis show smaller mitochondria, higher mitochondria membrane density, the vanishing of mitochondrial cristae, and the rupture of the mitochondrial outer membrane, which are different from apoptosis, necrosis, and autophagy in morphological features. Moreover, ferroptosis cannot be prevented by inhibitors of apoptosis, necrosis or autophagy (Dolma et al., 2003; Yang and Stockwell, 2008; Dixon et al., 2012). However, since the identification of ferroptosis, studies on the relationships among ferroptosis and other cell deaths have never stopped. Recent studies stated that ferroptosis shared a few common features with several types of cell death. Notably, although cells undergoing ferroptosis exhibit mitochondrial damage, ferroptotic cell death is not attributed to mitochondrial damage because the levels of ROS are unchanged in mitochondria in the cells treated with erastin. Moreover, ferroptosis also occurs in cells lacking the functional mitochondrial electron transport chain (ETC), the pathway that ROS are generated by in mitochondria (Dixon et al., 2012; Friedmann Angeli et al., 2014; Gaschler et al., 2018b).

### Ferroptosis and Oxytosis

Oxytosis is a type of oxidative cell death in neuronal cells. Oxytosis is induced by the glutamate-mediated inhibition of system x_c_^_^, which in turn leads to the depletion of GSH. The depletion of GSH damages the antioxidant defense of the cells and promotes the accumulation of ROS (Tan et al., 2001; Landshamer et al., 2008). The mechanism of glutamate-induced oxytosis appears to be the same as that of erastin-mediated ferroptosis. However, the protein BID which mediates mitochondrial integrity and function distinguishes ferroptosis from oxytosis (Landshamer et al., 2008; Grohm et al., 2010; Tobaben et al., 2011). Knockout of BID and the inhibitors of BID prevent both oxytosis and ferroptosis. Interestingly, the ferroptosis specific inhibitors, ferrostatin-1 and liproxstatin-1, also rescue glutamate-induced oxytosis and preserve mitochondrial integrity. The differences between ferroptosis and oxytosis are that ferroptosis cannot transactivate BID and that oxytosis does not share the indispensable AIF translocation with ferroptosis (Neitemeier et al., 2017). More potent evidence is needed to clarify the link between these two types of cell death.

### Ferroptosis and Necroptosis

Ferroptosis is distinct from apoptosis and necrosis, but reports showed that ferroptosis sometimes accompanied necroptosis. The neuronal cells death by hemorrhagic stroke simultaneously possesses features of ferroptosis and necroptosis. Inhibitors of ferroptosis (e.g., ferrostatin-1, deferoxamine, Trolox) and necroptosis (necrostatin-1) rescued hemoglobin- and hemin-induced toxicity, respectively. In addition, molecular markers of ferroptosis (phospho-ERK1/2) and mRNA levels of necroptosis markers (RIP1 and RIP3) were both increased in hemin-induced cell death. However, electron microscopy shows that cell death induced by hemin mainly exhibits necrotic morphology comprising a loss of plasma membrane integrity and the disruption of organelles with no observation of shrunken mitochondria, the distinguishing characteristic of ferroptosis (Zille et al., 2017). Another study also found that ferroptosis facilitated synchronized necrosis in renal tubules (Linkermann et al., 2014). In 2017, Tammo et al. found that ferroptosis and necroptosis are alternative forms of cell death. They used ACSL4 as the marker of sensitivity to ferroptosis and mixed lineage kinase domain-like (MLKL) as the marker of necroptosis. Interestingly, ACSL4 deficiency led to an increase in MLKL, and a loss of MLKL increased the ferroptosis-sensitivity of the cells. When one cell death pathway is inhibited, the other pathway is compensatorily enhanced (Muller et al., 2017). According to these results, Tonnus and Linkermann (2016) hypothesized that the free diffusion of NADPH between cells might account for the observation that necrosis undergoes synchronized regulation by ferroptosis in renal tubule cells: the regulation of necrosis also results in the depletion of NADPH and the loss of NADPH sensitizes cells to ferroptosis. The free diffusion of NADPH between adjacent cells causes them to tend to equilibrate in conditions with abundant NADPH. However, when cells suffer from necrosis or ferroptosis (i.e., either of which has a low abundance of NADPH), adjacent cells can more easily undergo another form of cell death (Tonnus and Linkermann, 2016). Although there is no explicit proof to test this hypothesis, it provides a likely model linking ferroptosis and other forms of cell death.

### Ferroptosis and Autophagy/Ferritinophagy

Autophagy is a progress in the cells that sequesters proteins and organelles in the autophagosomes and subsequently delivers them to lysosomes for degradation. Ferritinophagy is the autophagic process of ferritin, which is mediated by NCOA4. NCOA4 binds to FTH1 in the autophagosomes during low intracellular iron and the autophagosomes are then sent to lysosomes for the degradation of ferritin (Mancias et al., 2014). In senescent cells, iron was upregulated via the impaired ferritinophagy and inhibition of ferroptosis (Masaldan et al., 2018). In fibroblasts and cancer cells, the autophagy or ferritinophagy promoted ferroptosis by increasing the abundance of iron by the degradation of ferritin (Hou et al., 2016). Erastin-induced ferroptosis was coupled with the activation of ferritinophagy in liver fibrosis (Zhang et al., 2018). These reports stated that ferroptosis accompanied with the activation of ferritinophagy or ferritinophagy promoted the initiation of ferroptosis. Conversely, a study conducted in 2016 found that the inhibition of autophagy/ferritinophagy by its specific inhibitor and knockdown of NCOA4 prevented ferroptosis in mouse embryonic fibroblasts and HT1080 cells. This report stated that autophagy was required for the initiation of ferroptosis and that ferroptosis was an autophagic process (Gao et al., 2016). But what is the cargo receptor of ferroptosis and what is the substrate if ferroptosis is an autophagic cell death process?

In fact, we could also obtain an extra conclusion from this article: ferritinophagy might be a downstream process of ferroptosis. Furthermore, ferroptosis is different from ferritinophagy for it does not share common morphological characteristics with rapamycin-induced autophagy (e.g., the formation of double-membrane-enclosed vesicles) and specific inhibitors of autophagy cannot rescue ferroptosis (Dixon et al., 2012). Thus, we have assumed that ferritinophagy cannot be anything but a middle process of ferroptosis. But what is the final process in ferroptosis?

Taken together, the evidence shows that ferroptosis is different from other forms of cell death, but these various forms of cell deaths are not independent. The various forms of cell death are likely linking with each other to form a network to mediate cell availability. Studies on this network will certainly help us to better understand intractable diseases, such as tumorigenesis and IRI.

## Conclusion and Future Perspectives

Ferroptosis is a novel form of cell death with widespread functions in cells. Although many questions remain unclear, several facts are incontrovertible: (1) ferroptosis differs from other forms of cell death; (2) ferroptosis functions in a wide array of cells; (3) iron is required for ferroptosis; and (4) GPX4 inactivation is the key process in ferroptosis. Other issues, such as the roles of iron and lipid autoxidation, need more exploration. The accumulation of lipid ROS leads to ferroptosis, while it does not have a certain threshold value of ROS. It is likely that the ferroptosis-sensibility of the cells depends on the cell type, physiological conditions, and even individual life styles. Much more studies on the mechanism of ferroptosis are still required and a deeper understanding of ferroptosis will surely be beneficial to the treatment of relevant diseases.

## Author Contributions

All authors listed have made a substantial, direct and intellectual contribution to the work, and approved it for publication.

## Conflict of Interest Statement

The authors declare that the research was conducted in the absence of any commercial or financial relationships that could be construed as a potential conflict of interest.
